# Distinct Mindfulness States Produce Dissociable Effects on Neural Markers of Emotion Processing: Evidence From the Late Positive Potential

**DOI:** 10.1016/j.bpsgos.2024.100357

**Published:** 2024-06-29

**Authors:** Yanli Lin, Marne L. White, Deanna Wu, Natee Viravan, Todd S. Braver

**Affiliations:** a Department of Psychological and Brain Sciences, Washington University in St. Louis, St. Louis, Missouri; b Department of Psychiatry, Faculty of Medicine Siriraj Hospital, Mahidol University, Bangkok, Thailand

**Keywords:** Arousal, EEG, Emotion, ERP, Late positive potential, Mindfulness

## Abstract

**Background:**

Mindfulness has long been theorized to benefit emotion regulation, but despite the ubiquity of the claim, there is little empirical evidence demonstrating how mindfulness modulates the neurophysiology of emotion processing. The current study aimed to fill this gap in knowledge by leveraging a novel research approach capable of discretizing mindfulness into distinct states of open monitoring (OM) and focused attention (FA) to distinguish their influence on multimodal subjective and objective measures of emotion processing.

**Methods:**

Utilizing a fully within-participant picture viewing state induction protocol (*N* = 30), we compared the effects of OM and FA, rigorously contrasted against an active control, on the visually evoked late positive potential (LPP), a neural index of motivated attention. Bayesian mixed modeling was used to distinguish OM versus FA effects on the early and late sustained LPP while evaluating the influence of subjective arousal ratings as a within-participant moderator of the state inductions.

**Results:**

When negative picture trials were retrospectively rated as more subjectively arousing, the OM induction reduced the late sustained LPP response, whereas the FA induction enhanced the LPP.

**Conclusions:**

Acute manipulation of OM and FA states may reduce and enhance motivated attention to aversive stimuli during conditions of high subjective arousal, respectively. Functional distinctions between different mindfulness states on emotion processing may be most dissociable after accounting for within-participant variability in how stimuli are appraised. These results support the future potential of the state induction protocol for parsing the neural affective mechanisms that underlie mindfulness training programs and interventions.

## Plain Language Summary

Mindfulness, defined as a nonjudgmental awareness of present moment experience, is widely thought to benefit emotional well-being. Despite substantial research in this area, it remains unclear how mindfulness influences emotion-related processes at the level of brain functioning. The current study utilized an EEG picture viewing task to examine how open monitoring (OM) and focused attention (FA), 2 distinct mindfulness states, influence the late positive potential (LPP), a neural marker of motivated attention. The results show that when participants rated negatively valenced pictures as more subjectively arousing, the OM state decreased the LPP response, whereas the FA state increased the LPP. These findings suggest that OM and FA states may reduce and enhance motivated attention to unpleasant high arousing stimuli, respectively. At a broader level, the study shows that the effects of experientially distinct mindfulness states are distinguishable and provides a methodological foundation for future studies to disentangle the neural mechanisms of mindfulness programs and interventions.

Mindfulness, broadly defined as the nonjudgmental awareness and acceptance of present moment experience (1, 2, 3), has been widely theorized to confer salutary influences on emotion regulation (4, 5, 6), which is an essential self-regulatory ability that enables adaptive modulation of and responsivity to emotional experience (7,8). Despite substantial research interest in this domain, empirical evidence to fully support this view is lacking; in particular, it remains unclear exactly how mindfulness modulates emotion processing at the mechanistic neurophysiological level. Importantly, this critical gap in knowledge restricts thorough evaluation of the longstanding claim, and sometimes pervasive assumption, that mindfulness can be adopted and trained to benefit emotional well-being and mental health more broadly—a central promise driving public enthusiasm for mindfulness-related wellness programs and commodities (9). Moreover, mindfulness-based interventions have proliferated across psychiatric medicine (10) and are being increasingly utilized to treat mood and anxiety disorders for which emotion dysregulation is a hallmark feature (5,11, 12, 13). Thus, developing a critical understanding of whether and how mindfulness influences the neural underpinnings of basic affective functioning is an increasingly important scientific prerogative.

In the current study, we implemented a fully within-participant multisession electroencephalography (EEG) induction protocol to parse the distinct influences of open monitoring (OM) and focused attention (FA) on the visually evoked late positive potential (LPP), a well-investigated neural marker of emotion processing (14,15). The LPP’s amplitude is known to increase in correspondence with the arousal level of emotional stimuli (16,17) and has been demonstrably sensitive to a variety of emotion regulation strategies (18,19). Conversely, the LPP has been found to be disrupted in the context of emotion dysregulation, including among clinical populations suffering from mood, anxiety, and stress-related disorders (20, 21, 22). Notably, mindfulness and acceptance techniques have mostly been shown to reduce the LPP to negative high arousing images, indicating promotion of effective downregulation of emotion processing in response to aversive stimuli (23, 24, 25, 26, 27, 28), although null effects and LPP enhancement have also been reported (29,30).

Importantly, OM and FA are 2 distinct meditation practices that are collectively recognized as central but orthogonal training components of established mindfulness-based interventions (31): OM reflects a state of nonjudgmental awareness and acceptance of arising experience, whereas FA refers to goal-directed sustained attention on a target object (32). Critically, despite often being subsumed under the broad umbrella of mindfulness techniques, OM and FA may plausibly confer differential effects on emotion processing, which could adjudicate previous mixed findings involving mindfulness effects on the LPP (24,28, 29, 30) (see Discussion for elaboration). In particular, OM may attenuate the LPP through its selective promotion of attitudinal acceptance and interoceptive awareness, thereby facilitating an embodied “come and pass” experiential state to dampen the emotional saliency of negative images. Conversely, FA may enhance the LPP due to its strong emphasis toward sustaining attentional focus on the task at hand, which, in the context of an affective picture viewing paradigm, could prolong or even potentiate the emotionally evocative features of the target stimuli.

Within the overarching mindfulness construct, state mindfulness (e.g., OM vs. FA), trait mindfulness, and mindfulness training experience reflect central but poorly differentiated facets that may have distinct modulatory influences on the LPP. Moreover, we contend that it is particularly worthwhile to prioritize the investigation of state mindfulness because it reflects an operationalization of mindfulness that is inherently experiential and experimentally inducible via guided inductions [see (33) for a detailed review]. Equally critical, no mindfulness studies reported to date have sought to systematically investigate the interplay between the LPP and subjective arousal despite their longstanding linkage. Although stimuli arousal is one of the strongest and most reliable modulators of LPP amplitude (16,18,34,35), the LPP is also demonstratively sensitive to individual differences, contextual factors, and task manipulations that involve motivational significance and is thus widely thought to functionally reflect motivated attention processes [see (15,36,37) for reviews].

Therefore, the central innovation of the current study was to directly compare OM versus FA state effects on the LPP using a fully within-participant state induction protocol, modeling subjective picture viewing arousal ratings as a within-participant moderator while actively controlling for between-participant variability in trait mindfulness. Consequently, the explicit measurement and statistical modeling of subjective arousal in tandem with the LPP represents a significant opportunity to integrate subjective and objective approaches in thoroughly interrogating the affective properties of OM and FA states. Furthermore, the concordant assessment of average subjective arousal ratings, retrospectively obtained following the different state inductions, provides an experimental means through which to begin parsing the nature of the relationship between arousal and attention within the context of emotion processing. The results provide a proof-of-concept demonstration of our experimental approach to stimulate progress in this important domain.

## Methods and Materials

### Design and Procedures

The study protocol consisted of 3 random-order EEG testing sessions, each lasting approximately 2 hours and occurring on separate days, involving OM, FA, or active control (C) state inductions. Prior to beginning the first testing session, participants completed a self-report battery containing demographic information, trait mindfulness, and self-compassion questionnaires. After completion of EEG setup, each session began with a 10-minute guided audio induction of OM, FA, or C followed immediately by performance of the picture viewing or flanker task (task order randomized). After completing the first task, participants repeated the audio induction and then completed the second task (i.e., each participant completed the induction twice, prior to the start of each task, to ensure successful adoption and on-task maintenance of the state of interest). The sessions concluded with participants completing a brief manipulation check survey assessing engagement and responsivity to the induction and tasks, including subjective arousal ratings to negative and neutral images displayed during the picture viewing task. All participants completed the 3 sessions within a 1-week allotted time span. Additional description of the self-report measures is available online (https://osf.io/uv9yn/).

### Participants

Thirty native or fluent English-speaking novice participants with no mindfulness experience were recruited using flyers, email, and word-of-mouth advertising. One participant was excluded from all data analyses due to repeated failure to comply with task instructions (e.g., falling asleep or clicking randomly during tasks). Therefore, the final sample consisted of 29 participants (18–35 years old, mean = 20.72, SD = 4.04; 17 women, 12 men) totaling 87 sessions of data, which exceeded the preregistered target of 28 participants needed to detect the hypothesized within-participant interaction effects with 0.80 power (assuming a medium effect size of *d* = 0.50). Two participants did not complete the manipulation check measures during 1 session due to experimenter error (forgetting to administer the survey) and technical failure (the data collection server was down). Participants were compensated $80 for full completion of the study. The study protocol (202012148) was approved by Washington University in St. Louis’ Institutional Review Board.

### Preregistration

For full transparency, we submitted a preregistration outlining our a priori hypotheses and analytic approach [the submission also included analyses involving the flanker task that have already been reported in a separate manuscript; see (38)]. As alluded to above, on the basis of previous theoretical (31,39) and empirical (24,25,27,40) work, we hypothesized that the OM state induction would selectively reduce the LPP amplitude to negative high arousing stimuli compared with FA and active control conditions. Our preregistered analytic models yielded null results (the model outputs are openly available on our OSF repository), which consequently led us to conduct the current analyses that incorporated subjective arousal as a plausible moderator of the induction effects. The full preregistration is available at https://osf.io/2zk3s.

### Tasks

#### Audio Inductions

All audio inductions were exactly 10 minutes long. The OM induction instructed participants to direct awareness to arising thoughts, feelings, and bodily sensations in an open, nonjudgmental manner. The FA induction guided participants to sustain attention to their breath and redirect attention any time mind wandering occurred. Both mindfulness inductions were recorded by a certified Mindfulness-Based Stress Reduction instructor and used in previous work (38,41). The C induction was a duration-matched audio recording of a TED talk by the linguist Chris Lonsdale teaching participants how to rapidly acquire second language proficiency, which was also used in previous studies (24,42). All participants were instructed to keep their eyes open during the audio inductions to mitigate sleepiness among novice participants, which has been shown to modulate the LPP response (25).

#### Picture Viewing Task

Stimuli included 60 pictures, 30 negative (mean = 2.22) high arousing (mean = 6.63) pictures and 30 neutral (mean = 4.76) low arousing (mean = 3.20) pictures, from the International Affective Picture System [Lang *et al.* (43)], which were identical to what was used in previous work (24,25) and selected to maximize cross-study generalizability. The stimuli were presented using E-Prime software (Psychology Software Tools, Inc), which was used to control the timing and duration of picture onset/offset. Each trial began with a white fixation cross (+) at the center of a black screen for 500 ms. Then, a randomly selected picture was displayed on the entire screen for 5000 ms. The intertrial interval between picture offset and fixation onset varied randomly between 2000 and 4000 ms. The presentation of the 60 nonrepeating pictures were divided into 3 blocks of 20 trials, each block containing 10 negative and 10 neutral images.

Prior to the start of the task, participants were provided specific viewing instructions that matched each state induction (sustain attention on each picture for FA; attend nonjudgmentally to arising experience when viewing each picture for OM). Nonspecific viewing instructions were provided for the control condition (attend to each picture naturally). Importantly, during each of the 3 sessions, as part of the postsession questionnaire, participants retrospectively rated their arousal response (1 = not at all, 7 = very), giving one rating for the negative pictures and a separate rating for the neutral pictures presented during the task.

### Physiological Recording and Data Reduction

Participants were fit with a 64-channel lycra cap, and continuous EEG activity was recorded using a Brain Vision actiCHamp Plus system (Brain Vision LLC). Recordings were taken from 32 Ag-AgCl electrodes placed in accordance with the 10/20 system with a sampling rate of 500 Hz. Horizontal and vertical electrooculogram activity were recorded using 3 electrodes placed around the eyes, 2 lateral to the outer canthi of each eye and 1 directly under the right pupil and below electrode site Fp2.

Offline analyses were conducted using BrainVision Analyzer 2 (BrainProducts). All data were rereferenced to the average of all scalp electrodes (i.e., common average reference) and bandpass filtered between 0.01 and 20 Hz. Ocular artifacts were corrected using the regression approach developed by Gratton *et al.* (44). Artifact rejection was completed semiautomatically; a computer algorithm detected and flagged trials with at least 1 electrode that met the following criteria: a maximum voltage step of >50 μV between sample points, a voltage difference of >400 μV within 200-ms intervals, voltage exceeding ±200 μV, or a maximum voltage difference <0.5 μV within 1000-ms intervals. Trials containing 4 or more flagged electrodes or any artifacts detected at a midline site (Fpz, Fz, Cz, Pz, Oz) were removed.

As is standard, the LPP was time locked to picture onset with a 500-ms pretrial baseline correction and computed using the collapsed localizer method (45) at central-parietal recording site Pz, where amplitude was observed to be maximal. Replicating previous work (24,25), the early maximal LPP was quantified as the average activity occurring between 350 and 750 ms (±200 ms from which the LPP was maximally positive [550 ms]), whereas the late sustained LPP was quantified as the average voltage across 4 successive 1000 ms time windows ranging from 1000 to 5000 ms (i.e., 1000–2000 ms, 2000–3000 ms, etc.).

### Statistical Analyses and Predictions

The brms package in R statistical software was used to conduct Bayesian linear mixed-effects regression (46). Categorical variables (i.e., induction condition and picture type) were effect coded to enable proper estimation of main effects, while trait mindfulness composite score, session number, and task order were entered as continuous covariates. Time (1000–5000 ms separated across successive 1000-ms intervals) was entered as a continuous fixed effect to analyze the late sustained LPP. To aid interpretation, induction condition was also dummy coded when appropriate, with C or FA used as the referent condition to show how OM differed from C and FA. Trait mindfulness was computed as a normalized composite score by *z*-scoring the average of Five Facet Mindfulness Questionnaire (47) and Mindful Attention Awareness Scale (48) scores, as was done in previous work (38). Participant-level variability was modeled by entering a random intercept nested within participants.

Critical to the main aim of the exploratory analyses, we explicitly modeled within-participant–centered arousal ratings (computed by subtracting arousal values for each induction from the participant-level mean) as a fully interactive continuous predictor alongside induction condition (OM, FA, C) and picture type (negative, neutral), while also including an additional covariate that controlled for between-participant variability in arousal (computed by subtracting within-participant–centered arousal values from grand mean-centered arousal values). Moreover, because we did not detect any interactions involving time in the preregistered analysis, we removed time as a fully interactive variable to avoid modeling a 4-way interaction, electing to retain it as a continuous covariate instead. We were actively probing for a 3-way picture type × induction condition × arousal interaction to confirm our speculation that within-participant variability in subjective arousal ratings would moderate the influence of the state induction effects on the LPP.

## Results

Demographic characteristics of the sample are provided in Table 1, and descriptive statistics for the trait mindfulness measures and manipulation check items are provided in Tables 2 and 3, respectively. For brevity, we only describe effects that were relevant to testing the exploratory moderation among arousal ratings, induction condition, and picture type; a written description of the other effects, as well as additional reviewer suggested models, are available in the Supplement (https://osf.io/uv9yn/). Notably, the standard main effects of valence and time (larger LPP for negative than for neutral images and late LPP amplitude reducing across time intervals) were confirmed. Furthermore, there were no session or task order effects, assuaging concerns about habituation and unanticipated task order influences. These effects will not be described further below but will nevertheless be presented in the full model summaries presented in Tables 4 and 5.

**Table 1:** Demographic Characteristics of Participants

Variable	*n*
Gender	
Female	17
Male	12
Ethnicity	
Hispanic or Latino	2
Not Hispanic or Latino	27
Race	
American Indian or Alaskan Native	0
Asian	15
Black or African American	0
More than one race	0
Native Hawaiian or other Pacific Islander	1
White	13

**Table 2:** Descriptive Statistics for Mindfulness Measures (*n* = 29)

Variable	Range	Mean (SD)
FFMQ		
Total	78–177	123.86 (17.85)
Observe	18–37	25.72 (4.96)
Describe	17–35	27.69 (5.27)
Acting with awareness	11–37	23.69 (5.40)
Nonjudgment	12–40	25.45 (6.51)
Nonreactivity	10–31	21.31 (4.66)
MAAS	2.33–5.47	3.56 (0.75)

The FFMQ comprises 39 items (α = 0.91); The MAAS comprises 15 items (α = 0.87). Correlation between FFMQ total and MAAS: *r* = 0.53.

FFMQ, Five Facet Mindfulness Questionnaire; MAAS, Mindful Attention Awareness Scale.

**Table 3:** Descriptive Statistics for Manipulation Check Responses

Variable	C, *n* = 29		FA, *n* = 29		OM, *n* = 27	
	Range	Mean (SD)	Range	Mean (SD)	Range	Mean (SD)
Audio Engagement^a^	2–7	4.48 (1.60)	1–6	3.41 (1.38)	1–6	3.39 (1.34)
Audio Interest^a^	2–7	4.90 (1.52)	1–5	3.10 (1.05)	1–6	3.14 (1.38)
Audio Emotional Reaction^a^	3–7	4.93 (1.13)	1–6	4.17 (1.17)	3–7	4.39 (0.99)
Audio Arousal^a^	1–6	3.38 (1.78)	1–5	2.45 (1.21)	1–5	2.56 (1.25)
Audio Understanding^a^	3–7	5.97 (1.09)	5–7	6.48 (0.69)	2–7	5.63 (1.28)
Audio Learning^a^	2–7	4.93 (1.49)	1–6	3.66 (1.29)	1–7	4.41 (1.45)
Audio Physical Comfort^a^	3–7	5.17 (1.20)	2–6	4.38 (1.15)	3–6	4.56 (1.12)
Audio Sleepiness	1–6	3.52 (1.60)	1–6	4.14 (1.53)	1–7	4.11 (1.62)
PANAS - Negative Scale	10–23	13.69 (3.90)	10–29	13.69 (4.43)	10–20	13.79 (3.12)
Picture Engagement	2–7	4.45 (1.27)	2–7	4.62 (1.47)	1–7	4.39 (1.45)
Picture Interest	2–7	4.07 (1.13)	1–7	4.07 (1.56)	1–7	4.14 (1.43)
Neutral Picture Arousal	1–5	2.45 (1.21)	1–5	2.52 (1.24)	1–5	2.39 (1.23)
Negative Picture Arousal	1–7	4.86 (1.87)	1–7	5.00 (1.54)	1–7	4.56 (1.53)
Picture Emotional Reaction	2–5	3.38 (0.94)	1–5	3.38 (0.90)	2–5	3.59 (0.64)
Picture Arousal Overall	1–7	4.07 (1.39)	1–7	4.28 (1.46)	1–7	4.00 (1.39)
Picture Physical Comfort	3–7	4.35 (1.14)	3–7	4.52 (1.02)	3–6	4.52 (0.98)
Picture Sleepiness	1–8	2.93 (1.98)	1–5	2.52 (1.38)	1–7	3.07 (1.63)

C, control; FA, focused attention; OM, open monitoring; PANAS, Positive and Negative Affect Schedule.

a Significant difference by condition (*F*s > 4.27, *p*s < .19).

**Table 4:** Model Output for Early LPP Exploratory Linear Regression Analysis

Model	Fixed Effects	Estimate (SD)	95% CI	R-hat	Bulk ESS	Tail ESS
Early LPP	Intercept	4.71 (2.03)	0.70 to 8.69^a^	1.00	1376	1895
	Picture type	1.61 (0.24)	1.13 to 2.08^a^	1.00	3038	3015
	Induction FA	0.39 (0.35)	−0.30 to 1.05	1.00	2534	2879
	Induction OM	−0.17 (0.35)	−0.87 to 0.51	1.00	2845	3094
	Induction C	−0.21 (0.34)	−0.88 to 0.44	1.00	2349	2582
	Within-participant arousal	0.03 (0.16)	−0.28 to 0.34	1.00	2735	2793
	Between-participant arousal	−0.06 (0.54)	−1.16 to 1.00	1.00	1593	2040
	Trait mindfulness	0.24 (0.61)	−0.99 to 1.40	1.00	1659	2278
	Session number	−0.15 (0.19)	−0.52 to 0.22	1.00	4189	2815
	Task number	0.44 (1.22)	−1.96 to 2.90	1.00	1370	1788
	Picture type × induction FA	0.38 (0.35)	−0.30 to 1.09	1.00	2644	3080
	Picture type × induction OM	−0.35 (0.35)	−1.07 to 0.35	1.00	2553	2649
	Picture type × induction C	−0.03 (0.34)	−0.70 to 0.64	1.00	2256	2696
	Picture type × within-participant arousal	0.07 (0.20)	−0.32 to 0.46	1.00	3358	2858
	Induction FA × within-participant arousal	−0.22 (0.24)	−0.68 to 0.25	1.00	2565	2544
	Induction OM × within-participant arousal	0.21 (0.25)	−0.27 to 0.72	1.00	2458	2921
	Induction C × within-participant arousal	0.01 (0.23)	−0.44 to 0.46	1.00	2204	2432
	Picture type × induction FA × within-participant arousal	−0.26 (0.23)	−0.70 to 0.19	1.00	2600	2784
	Picture type × induction OM × within-participant arousal	0.06 (0.24)	−0.43 to 0.53	1.00	2829	2813
	Picture type × induction C × within-participant arousal	0.21 (0.22)	−0.24 to 0.65	1.00	2580	2795

Picture type is negative. Within-participant arousal was computed by subtracting arousal values for each induction from the participant-level mean; between-participant arousal was computed by subtracting within-participant–centered arousal values from grand mean-centered arousal values.

C, control; ESS, effective sample size; FA, focused attention; LPP, late positive potential; OM, open monitoring.

a 95% CI does not contain 0.

**Table 5:** Model Output for Late LPP Exploratory Linear Regression Analysis

Model	Fixed Effects	Estimate (SD)	95% CI	R-hat	Bulk ESS	Tail ESS
Late LPP	Intercept	−0.45 (1.09)	−2.59 to 1.72	1.00	1401	2463
	Picture type	0.53 (0.15)	0.22 to 0.83^a^	1.00	2910	2871
	Induction FA	−0.11 (0.22)	−0.54 to 0.32	1.00	2464	2697
	Induction OM	0.40 (0.22)	−0.06 to 0.83	1.00	2473	2550
	Induction C	−0.29 (0.22)	−0.72 to 0.13	1.00	2454	2573
	Within-participant arousal	0.16 (0.10)	−0.05 to 0.36	1.00	2932	3112
	Between-participant arousal	−0.06 (0.30)	−0.65 to 0.53	1.00	1614	2152
	Time	−0.41 (0.08)	−0.57 to −0.24^a^	1.00	4811	2851
	Trait mindfulness	0.54 (0.31)	−0.08 to 1.17	1.00	1745	2237
	Session number	0.13 (0.12)	−0.10 to 0.36	1.00	4021	2752
	Task number	0.91 (0.64)	−0.37 to 2.18	1.00	1235	2155
	Picture type × induction FA	−0.30 (0.23)	−0.75 to 0.14	1.00	2578	2965
	Picture type × induction OM	0.15 (0.23)	−0.28 to 0.59	1.00	2045	2706
	Picture type × induction C	0.16 (0.22)	−0.26 to 0.59	1.00	2643	2799
	Picture type × within-participant arousal	0.36 (0.13)	0.11 to 0.62^a^	1.00	3637	2804
	Induction FA × within-participant arousal	0.40 (0.15)	0.10 to 0.70^a^	1.00	2505	2602
	Induction OM × within-participant arousal	−0.23 (0.16)	−0.55 to 0.09	1.00	2130	2970
	Induction C × within-participant arousal	−0.17 (0.14)	−0.46 to 0.10	1.00	2603	2965
	Picture type × induction FA × within-participant arousal	0.35 (0.15)	0.06 to 0.63^a^	1.00	2364	2875
	Picture type × induction OM × within-participant arousal	−0.53 (0.15)	−0.82 to −0.22^a^	1.00	2470	2550
	Picture type × induction C × within-participant arousal	0.18 (0.14)	−0.10 to 0.46	1.00	2234	2605

Picture type is negative. Within-participant arousal was computed by subtracting arousal values for each induction from the participant-level mean; between-participant arousal was computed by subtracting within-participant–centered arousal values from grand mean-centered arousal values.

C, control; ESS, effective sample size; FA, focused attention; LPP, late positive potential; OM, open monitoring.

a 95% CI does not contain 0.

### Early LPP

Subjective arousal ratings did not moderate the influence of the state inductions on the early LPP (*b*s < |0.26|, all CIs contain 0), and there were no main or interaction effects involving induction condition, demonstrating that the state inductions did not modulate the early LPP at all (*b*s < |0.38|, all CIs contain 0) (see Table 4).

### Late LPP

**Figure 1:**
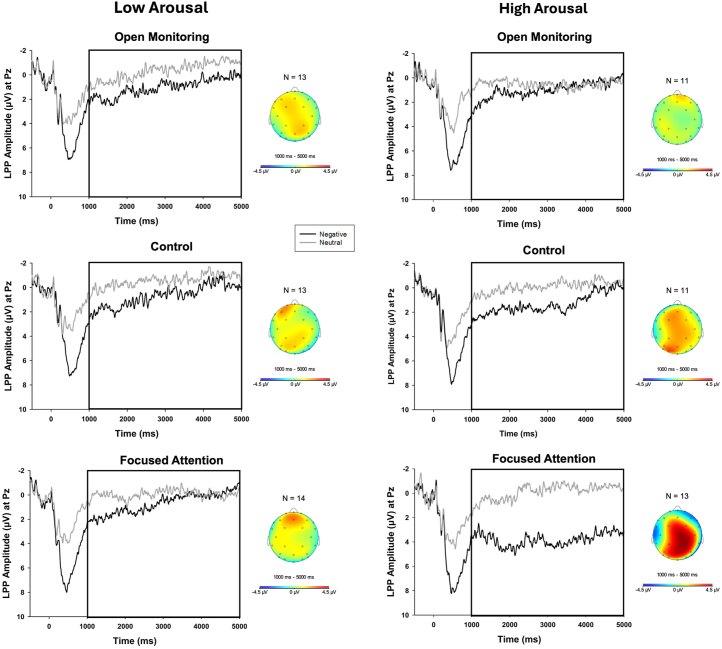
Late positive potential (LPP) waveforms and topographical head map for participants reporting higher within-participant arousal ratings on negative picture trials, separated across induction condition (selected via median split for each induction). Grand average waveforms are computed by averaging each participant’s waveforms across negative (dark line) and neutral (gray line) trials at electrode site Pz and then averaging across group (number of averaged participants are reported across arousal level and induction condition, excluding participants at the median). Time 0 represents the onset of the stimulus. Head map provides scalp topography of difference in response amplitude between negative and neutral trials across the bracketed 1000–5000 ms time window. As visualized, open monitoring produced attenuated LPPs compared with both control (*b* = −0.71; SD = 0.26; 95% CI, −1.22 to −0.20; evidence ratio = 234.29) and focused attention (*b* = −0.87; SD = 0.25; 95% CI, −1.35 to −0.37; evidence ratio = 3999) at higher levels of arousal.

Supporting the widely held but rarely tested assumption, when participants reported higher arousal ratings to the negative pictures (than neutral pictures), these were decisively associated with larger LPP amplitudes (i.e., arousal × picture type interaction; *b* = 0.36; SD = 0.13; 95% CI, 0.11 to 0.62; evidence ratio [ER] = 332.33). Critically, this interaction was further qualified by a 3-way arousal × picture type × induction interaction. Notably, higher negative picture arousal ratings during the OM induction were selectively associated with smaller LPP responses to negative pictures (*b* = −0.53; SD = 0.15; 95% CI, −0.82 to −0.22; ER = 1999), whereas higher arousal during the FA induction was associated with larger negative trial LPPs (*b* = 0.35; SD = 0.15; 95% CI, 0.06 to 0.63; ER = 110.11). Completing this dissociable pattern, higher arousal to negative pictures during the C induction was unrelated to the LPP (*b* = 0.18; SD = 0.14; 95% CI, −0.10 to 0.46; ER = 0.13). Dummy-coded contrasts clearly demonstrated that higher negative picture arousal ratings during OM were associated with attenuated LPPs compared with both C (*b* = −0.71; SD = 0.26; 95% CI, −1.22 to −0.20; ER = 234.29) and FA (*b* = −0.87; SD = 0.25; 95% CI, −1.35 to −0.37; ER = 3999) inductions. Conversely, higher negative picture arousal ratings during FA were associated with larger LPPs than OM (*b* = 0.87; SD = 0.26; 95% CI, 0.36 to 1.39; ER = 1999) but not C (*b* = 0.16; SD = 0.24; 95% CI, −0.30 to 0.64; ER = 3.04). A full summary of the model output is presented in Table 5 together with visualization of the key interaction involving arousal, picture type, and induction condition depicted in Figure 1.

## Discussion

The current study leveraged a within-participant state induction protocol to directly compare the effects of OM and FA on the early and late LPP. An initial set of preregistered analyses testing the hypothesis that OM would selectively reduce the LPP yielded null results, prompting us to conduct follow-up exploratory analyses to examine the moderating influence of retrospective picture arousal ratings collected at the end of each induction session. Consistent with previous work, we found no induction-related effects on the early LPP, supporting previous postulations that brief state mindfulness manipulations, at least in novice samples, may be insufficient to modulate early bottom-up attentional mechanisms during picture viewing (24,25). In contrast, more stable dispositional indices of mindfulness such as meditation experience and trait mindfulness have both been linked to reduced early LPP amplitudes (23,26). Regarding the late sustained LPP, however, our models revealed that the OM induction reduced the LPP response to negative pictures, whereas the FA induction enhanced the LPP, but only when incorporating retrospective subjective arousal ratings. Specifically, our a priori predicted effects were not significant in the original preregistered models but instead were present when negative picture trials were rated as more subjectively arousing.

Critically, these results provide, to the best of our knowledge, the first direct empirical demonstration that theoretically distinct meditation states exert functionally distinguishable effects on the LPP, insofar as OM and FA may reduce and increase the motivational significance/salience of aversive emotionally evocative stimuli, respectively (15). Notably, these contrasts were preferentially identified under relative conditions of higher retrospective subjective arousal, illustrating that the psychological and neural distinctions between OM and FA may be most dissociable after explicitly measuring and accounting for variability in how the pictures were experienced and appraised. Moreover, the observation that induction effects on LPP amplitude differed systematically as a function of arousal, despite no mean differences in arousal ratings across inductions, introduces the promise of disentangling attention and affect from the broader context of emotion processing, suggesting that state mindfulness (i.e., both OM and FA) may more strongly modulate motivated attention mechanisms as opposed to directly reducing subjective arousal as is sometimes presumed. This interpretation is remarkably compatible with leading functional theories of the LPP, which posit that its amplitude reflects visual attention gating mechanisms that are contingent upon the motivational significance of the stimuli (15,36).

Importantly, our findings also provide a new and promising empirical basis from which to extend and disambiguate previous findings. Toward this end, the OM effect observed here is largely consistent with past studies that have linked brief OM state inductions with reduced LPPs to negative high arousing stimuli in both adults (24,25,27) and children (49). On the other hand, our FA results may help clarify past outcomes that were previously unexpected or construed as contradictory. For example, Egan *et al.* reported an increase in the LPP across all picture types after a brief state mindfulness manipulation (29), whereas Zhang *et al.* observed reduced LPP amplitudes (28). Upon closer examination, Egan *et al.* utilized state mindful viewing instructions that were nearly identical to those of our FA condition (i.e., explicitly instructing sustained attention to each picture), reinforcing the possibility that instructional application of FA during picture viewing may modulate motivated attention processes to globally enhance the LPP—a pattern that was actually evidenced in the current dataset (see the 2-way induction × arousal interaction in Table 5). Conversely, viewing instructions in the study by Zhang *et al.* (28) shared more similarities with our OM induction, which likewise directed attention internally toward arising psychological and bodily experiences as opposed to externally toward the pictures themselves. Together, these considerations support the overarching notion that differences in the scope and object of awareness (i.e., internal vs. external attention allocation) may be critical factors that distinguish OM versus FA effects on the direction and magnitude of the LPP response.

From a methodological perspective, our study demonstrates the promise of explicitly linking subjective and objective task measures using a fully within-participant design, showing that functional distinctions between OM and FA states may be more salient and observable when key subjective features of picture viewing (self-reported arousal) are directly assessed and modeled in tandem with standard objective neural metrics (LPP amplitude). As we have reported previously, investigations aimed at distinguishing the influence of different meditation states and practices may hold greater conceptual clarity, methodological precision, and ecological validity when conducted using within- as opposed to between-participant designs [see (38)]. With the current design and analyses, which drew inspiration from other within-participant approaches from the broader emotion regulation and LPP literature [e.g., (50,51)], we were also able to test the criterion validity of the picture viewing task itself, confirming that larger differences in the magnitude of the LPP response for negative than for neutral pictures were associated with higher self-reported arousal across the 2 picture types. Therefore, all state induction effects were detected after explicitly verifying and accounting for this frequently assumed, but rarely tested, relationship.

Despite these conceptual and methodological advances, our study is not without limitations. First, it must be sharply cautioned that the analyses conducted here are exploratory and that the sample size is small, only marginally exceeding the minimum threshold of participants computed from our power analysis. Although most of the design and analytic considerations were preregistered, the modeling of picture arousal ratings was not preplanned; therefore, our results cannot be interpreted as confirmatory and remain in need of replication. As mentioned in our previous work (52), our analytic procedures are tailored for Bayesian updating to accommodate dedicated follow-up efforts through which exact parameter values and effect sizes can be tested against new data until sufficient evidence is obtained (53, 54, 55).

Second, our measure of picture arousal was constrained in time, frequency, and modality to 2 self-report questionnaire items (negative and neutral) administered retrospectively at the end of each testing session rather than being acquired on a trial-by-trial basis. Likewise, the LPP was quantified using the well-established collapsed localizer method (45), which reduced signal noise at the expense of capturing trial-level variability. Consequently, the temporal coarseness and restricted means of measurement limit inferences regarding the nature, significance, and underlying dynamics of the relationship between arousal and the LPP. A more powerful study design would be to incorporate a multimodal trial-by-trial approach, embedding passive measures of arousal (e.g., galvanic skin conductance, pupil dilation) in tandem with LPP quantification and self-report arousal ratings at the single trial level. Relatedly, although instructions were provided to experimentally manipulate state engagement during task performance, the extent to which participants were able to follow direction remains fundamentally unclear. Therefore, inclusion of visual attention measures (e.g., eye tracking) could offer a more direct method to test for instructional compliance, while potentially extending understanding of the emergent possibility that OM and FA may differentially modulate the LPP via internal versus external biasing of attention.

### Conclusions

In conclusion, the current investigation exemplified the promise of using a fully within-participant experimental approach to parse the psychological and neural effects of putatively distinct mindfulness states and practices on emotion processing. Although encouraging, these findings are fundamentally exploratory and should be considered primarily as a proof-of-concept research approach. Nevertheless, the results represent a first step toward elucidating how different facets of the mindfulness construct influence emotion processes across the interactive levels of mind, brain, and behavior. Toward this end, we hope that our study stimulates the collaborative interest of other researchers to make further progress together, ideally in extending these basic findings toward translational and clinical applicability. Developing a clearer mechanistic understanding of mindfulness may be integral toward fully harnessing and optimizing its salutary potential to benefit those who are most in need.

## Supplementary Material

Key Resources Table
