# Supplementary material for: Distinct Mindfulness States Produce Dissociable Effects on Neural Markers of Emotion Processing: Evidence From the Late Positive Potential
Source: Biol Psychiatry Glob Open Sci. 2024 Jun 29;4(5):100357. doi: 10.1016/j.bpsgos.2024.100357 (PMC11342101; doi:10.1016/j.bpsgos.2024.100357)
Supplement: Supplementary Information [file mmc2.pdf]

SUPPLEMENTARY INFORMATION

Distinct Mindfulness States Produce Dissociable Effects on Neural Markers of Emotion Processing: Evidence From the Late Positive Potential

Lin *et al.*

Self-report Measures

Participants completed two trait mindfulness measures, the 39-item Five Facet Mindfulness Questionnaire (FFMQ; 1) and the 15-item Mindful Attention Awareness Scale (MAAS; 2); as well as a manipulation check administered at the end of each session to assess for potential differences in engagement and receptivity to the audio inductions and tasks. Participant response patterns on the manipulation check battery are reported below.

Description of manipulation check responses

One-way repeated measures ANOVAs and follow-up Bonferroni corrected t-tests were conducted to assess for session differences on the manipulation check measures. Participant responses to the guided inductions have already been reported previously (3). Briefly, participants rated the active control session as more engaging, interesting, and physically comfortable than both FA and OM. Participants also reacted more positively to C than FA and reported higher levels of arousal during C relative to OM. Lastly, participants endorsed higher levels of understanding during FA compared to both C and OM (see 3 for full output).

For the picture viewing task, participants reported their engagement, interest, and arousal to both negative and neutral pictures, in addition to their overall arousal (*1 = not at all, 7 = very*), emotional reactivity (*1 = very negative, 4 = neutral, 7 = very positive*), and physical comfort (*1 = not comfortable, 7 = very comfortable*) during the task. Participants also reported their negative affect by rating the extent to which they were distressed, upset, guilty, scared, hostile, irritable, ashamed, nervous, jittery, and afraid (*1 = very slightly or not at all, 3 = moderately, 5 = extremely*) using the Negative Affect Schedule (NAS; 4). Lastly, participants rated their sleepiness level (*1 = feeling active, vital, alert, or wide awake*, *8 = I fell asleep*) using the Stanford Sleepiness Scale

(SSS; 5). There were no significant session differences across any of these items (*F*s < 1.28, *p*s > 0.29).

Bayesian model specifications and statistical reporting

The models were run using 4 Monte Carlo chains, each containing 2,000 iterations with 1,000 warm-up iterations which were discarded. Given the exploratory nature of the analysis, uniform priors were conservatively selected to estimate fixed effects across all models, and random effects were weakly informative based on *brms* defaults. We report the mean, standard deviation (SD), 95% credible interval (CI), R-hat and ESS values for all parameter estimates. We also computed the evidence ratio (ER) to directly weigh evidence in favor or against expected interactions involving arousal ratings, picture type, and induction condition.

Additional description of manuscript results

Early LPP

As standard, there was a main effect of Picture Type, such that negative pictures were associated with larger early LPP amplitudes relative to neutral pictures (*b* = 1.61, *sd* = 0.24, 95% CI = [1.13, 2.08]). There were no other significant main or interactive effects (*b*s < |0.44|, all 95% CIs contain 0).

Late Sustained LPP

There were main effects of Picture Type and Time, such that negative pictures were associated with larger late LPP amplitudes relative to neutral pictures (*b* = 0.53, *sd* = 0.15, 95% CI = [0.22, 0.83]); and that the late LPP decreased over time (*b*= -0.41, *sd* = 0.08, 95% CI = [-0.57, -0.24]). There were no other main effects (*b*s < |0.91|, all 95% CIs contain 0). All significant interactions are reported in the results section of the manuscript.

Reviewer requested supplemental analyses and model output

Model 1: Moderation by Negative Affect

Early LPP

There was a main effect of Picture Type, such that negative pictures were associated with larger early LPP amplitudes relative to neutral pictures (*b* = 1.64, *sd* = 0.15, 95% CI = [1.35, 1.93]). There were no other main or interactive effects (*b*s < |0.61|, all 95% CIs contain 0). See Table 1a for full output.

Late LPP

There were main effects of Picture Type, Time, and Trait Mindfulness, such that negative pictures were associated with larger late LPP amplitudes relative to neutral pictures (*b* = 0.76, *sd* = 0.09, 95% CI = [0.58, 0.94]); the late LPP decreased over time (*b* = -0.41, *sd* = 0.08, 95% CI = [-0.57, -0.25]); and that participants with higher levels of trait mindfulness exhibited larger LPPs collapsed across picture type (*b* = 0.79, *sd* = 0.29, 95% CI = [0.20, 1.38]). There was also a main effect of induction such that FA was associated with larger LPPs (*b* = 0.36, *sd* = 0.13, 95% CI = [0.11, 0.62]), but OM was associated with smaller LPPs (*b* = -0.26, *sd* = 0.13, 95% CI = [-0.51, -0.01]). Further, there were main effects of Negative Affect, such that participants with higher levels of within- (*b* = 0.11, *sd* = 0.04, 95% CI = [0.02, 0.19]) and between-subject negative affect (*b* = 0.29, *sd* = 0.11, 95% CI = [0.08, 0.50]) exhibited larger LPPs. The main effect of Within-Subject Negative Affect was further qualified by two-way Picture Type x Negative Affect and Induction Condition x Negative Affect interactions. First, participants who reported increased negative affect had larger LPPs on negative picture trials (*b* = 0.13, *sd* = 0.04, 95% CI = [0.05, 0.20]). Second, participants with higher self-reported negative affect during FA had larger LPPs collapsed across picture type (*b* = 0.17, *sd* = 0.07, 95% CI = [0.04, 0.31]), whereas participants reporting higher

negative affect during C exhibited smaller LPPs (*b* = -0.13, *sd* = 0.07, 95% CI = [-0.26, -0.01]) These two-way interactions were further qualified by a three-way Picture Type x Induction x Negative Affect interaction. Participants with higher self-reported negative affect during FA had larger LPPs on negative picture trials (*b* = 0.27, *sd* = 0.05, 95% CI = [0.18, 0.36]), but smaller negative LPPs during OM (*b* = -0.12, *sd* = 0.06, 95% CI = [-0.23, -0.01]) and C (*b* = -0.16, *sd* = 0.05, 95% CI = [-0.25, -0.06]). There were no other main or interactive effects (*b*s < |1.05|, all 95% CIs contain 0). See Table 1b for full model output.

Table 1a. *Model output of negative affect moderation for early LPP*

Model	Fixed Effects	Estimate (SD)	95% CI	R-hat	Bulk ESS	Tail ESS
Early LPP	Intercept	4.70 (1.93)	[0.93, 8.54]*	1.00	1074	1694
	Pic Type	1.64 (0.15)	[1.35, 1.93]*	1.00	3843	2655
	Induction FA	0.05 (0.21)	[-0.36, 0.47]	1.00	3022	2633
	Induction OM	-0.10 (0.21)	[-0.50, 0.30]	1.00	2867	2869
	Induction C	0.05 (0.21)	[-0.37, 0.46]	1.00	3001	2715
	Within-Sub Negative Affect	-0.06 (0.07)	[-0.20, 0.08]	1.00	2983	2545
	Between-Sub Negative Affect	0.38 (0.22)	[-0.03, 0.81]	1.00	1133	1762
	Trait Mindfulness	0.61 (0.60)	[-0.56, 1.75]	1.00	1193	1611
	Session Number	-0.20 (0.20)	[-0.60, 0.19]	1.00	3630	2745
	Task Number	0.56 (1.15)	[-1.72, 2.81]	1.00	1069	1839
	Pic Type:Induction FA	0.10 (0.20)	[-0.31, 0.50]	1.00	3649	3141
	Pic Type:Induction OM	-0.09 (0.21)	[-0.50, 0.31]	1.00	3679	3225
	Pic Type:Induction C	-0.01 (0.21)	[-0.41, 0.41]	1.00	3923	3129
	Pic Type:Within-Sub Negative Affect	-0.01 (0.06)	[-0.13, 0.10]	1.00	3554	3011
	Induction FA:Within-Sub Negative Affect	0.03 (0.11)	[-0.19, 0.26]	1.00	2710	2500
	Induction OM:Within-Sub Negative Affect	-0.06 (0.12)	[-0.30, 0.16]	1.00	2859	2974
	Induction C: Within-Sub Negative Affect	0.03 (0.11)	[-0.19, 0.24]	1.00	3000	2934
	Pic Type:Induction FA:Within-Sub Negative Affect	-0.06 (0.08)	[-0.22, 0.09]	1.00	3182	2606
	Pic Type:Induction OM: Within-Sub Negative Affect	0.10 (0.10)	[-0.09, 0.30]	1.00	2948	2866
	Pic Type:Induction C:Within-Sub Negative Affect	-0.04 (0.08)	[-0.19, 0.11]	1.00	3136	2922

Note. Pic Type = Negative, FA = Focused Attention, OM = Open Monitoring, C = Control.
* denotes 95% CI does not contain 0.

Table 1b. *Model output of negative affect moderation for late LPP*

Model	Fixed Effects	Estimate (SD)	95% CI	R-hat	Bulk ESS	Tail ESS
Late LPP	Intercept	-0.27 (0.98)	[-2.16, 1.66]	1.00	1310	1923
	Pic Type	0.76 (0.09)	[0.58, 0.94]*	1.00	5502	3215
	Induction FA	0.36 (0.13)	[0.11, 0.62]*	1.00	4836	3247
	Induction OM	-0.26 (0.13)	[-0.51, -0.01]*	1.00	4463	3421
	Induction C	-0.11 (0.13)	[-0.35, 0.15]	1.00	3937	2958
	Within-Sub Negative Affect	0.11 (0.04)	[0.02, 0.19]*	1.00	3565	3275
	Between-Sub Negative Affect	0.29 (0.11)	[0.08, 0.50]*	1.00	1101	1817
	Trait Mindfulness	0.79 (0.29)	[0.20, 1.38]*	1.00	1487	2126
	Time	-0.41 (0.08)	[-0.57, -0.25]*	1.00	5362	2874
	Session Number	0.17 (0.13)	[-0.08, 0.42]	1.00	3943	2634
	Task Number	1.05 (0.56)	[-0.05, 2.15]	1.02	1229	1707
	Pic Type:Induction FA	0.24 (0.13)	[-0.01, 0.49]	1.00	3929	2979
	Pic Type:Induction OM	-0.15 (0.13)	[-0.41, 0.11]	1.00	4008	3218
	Pic Type:Induction C	-0.08 (0.13)	[-0.33, 0.18]	1.00	3929	3309
	Pic Type:Within-Sub Negative Affect	0.13 (0.04)	[0.05, 0.20]*	1.00	4956	2976
	Induction FA:Within-Sub Negative Affect	0.17 (0.07)	[0.04, 0.31]*	1.00	2610	2548
	Induction OM:Within-Sub Negative Affect	-0.04 (0.08)	[-0.19, 0.11]	1.00	2883	2743
	Induction C: Within-Sub Negative Affect	-0.13 (0.07)	[-0.26, -0.01]*	1.00	2723	2921
	Pic Type:Induction FA:Within-Sub Negative Affect	0.27 (0.05)	[0.18, 0.36]*	1.00	3617	3036
	Pic Type:Induction OM: Within-Sub Negative Affect	-0.12 (0.06)	[-0.23, -0.01]*	1.00	3266	3031
	Pic Type:Induction C:Within-Sub Negative Affect	-0.16 (0.05)	[-0.25, -0.06]*	1.00	3680	3275

Note. Pic Type = Negative, FA = Focused Attention, OM = Open Monitoring, C = Control.
* denotes 95% CI does not contain 0.

Model 2: Moderation by Self-Compassion

Early LPP

There was a main effect of Picture Type, such that negative pictures were associated with larger early LPP amplitudes relative to neutral pictures (b = 1.65, sd = 0.14, 95% CI = [1.38, 1.94]). There was also a main effect of Self-Compassion, such that participants with higher levels of self-compassion had smaller LPPs collapsed across picture type (b = -1.53, sd = 0.53, 95% CI = [-2.56, -0.51]). There were no other main or interactive effects (bs < |0.86|, all 95% CIs contain 0). See Table 2a for full model output.

Late LPP

There were main effects of Picture Type and Time, such that negative pictures were associated with larger late LPP amplitudes relative to neutral pictures (b = 0.79, sd = 0.09, 95% CI = [0.60, 0.97]); and that the late LPP decreased over time (b = -0.41, sd = 0.08, 95% CI = [-0.58, -0.25]). There was also a main effect of Induction, such that FA was associated with larger LPPs collapsed across picture type (b = 0.36, sd = 0.14, 95% CI = [0.09, 0.62]). The main effect of Picture Type was qualified by a two-way Picture Type x Self-Compassion interaction, such that participants with higher levels of self-compassion exhibited larger LPPs on negative picture trials (b = 0.21, sd = 0.09, 95% CI = [0.03, 0.38]). This two-way interaction was further qualified by a three-way Self-Compassion x Picture Type x Induction interaction. Specifically, participants with higher levels of self-compassion exhibited larger LPPs on negative picture trials during FA (b = 0.66, sd = 0.13, 95% CI = [0.40, 0.92]) but smaller negative LPPs during both the C (b = -0.33, sd = 0.14, 95% CI = [-0.60, -0.08]) and OM inductions (b = - 0.33, sd = 0.14, 95% CI = [-0.60, -0.06]). There were no other main or interactive effects (bs < |0.83|, all 95% CIs contain 0). See Table 2b for full model output.

**Table 2a.** *Model output of self-compassion moderation for early LPP*						
**Model**	**Fixed Effects**	**Estimate (SD)**	**95% CI**	**R-hat**	**Bulk ESS**	**Tail ESS**
Early LPP	Intercept	4.91 (1.70)	[1.66, 8.58]*	1.00	1296	1520
	Pic Type	1.65 (0.14)	[1.38, 1.94]*	1.00	3911	2889
	Induction FA	0.07 (0.20)	[-0.32, 0.47]	1.00	3779	3131
	Induction OM	-0.12 (0.20)	[-0.51, 0.28]	1.00	3616	3137
	Induction C	0.05 (0.21)	[-0.35, 0.45]	1.00	3228	3046
	Self-Compassion	-1.53 (0.53)	[-2.56, -0.51]*	1.00	1415	1973
	Trait Mindfulness	0.86 (0.56)	[-0.25, 1.93]	1.00	1531	2217
	Session Number	-0.11 (0.18)	[-0.47, 0.24]	1.00	4005	2674
	Task Number	0.30 (1.02)	[-1.89, 2.26]	1.00	1258	1620
	Pic Type:Induction FA	0.09 (0.21)	[-0.30, 0.50]	1.00	3447	3032
	Pic Type:Induction OM	-0.08 (0.20)	[-0.47, 0.32]	1.00	3341	2931
	Pic Type:Induction C	-0.02 (0.20)	[-0.44, 0.37]	1.00	3881	3013
	Pic Type: Self-Compassion	-0.18 (0.15)	[-0.46, 0.11]	1.00	4132	2765
	Induction FA: Self-Compassion	-0.08 (0.21)	[-0.49, 0.34]	1.00	3435	2692
	Induction OM: Self-Compassion	0.25 (0.21)	[-0.15, 0.66]	1.00	3157	2990
	Induction C: Self-Compassion	-0.17 (0.20)	[-0.58, 0.23]	1.00	2849	3038
	Pic Type:Induction FA: Self-Compassion	0.10 (0.21)	[-0.31, 0.53]	1.00	3690	3031
	Pic Type:Induction OM: Self-Compassion	-0.05 (0.21)	[-0.46, 0.36]	1.00	3767	3199
	Pic Type:Induction C: Self-Compassion	-0.06 (0.21)	[-0.48, 0.35]	1.00	3319	3111

Note. Pic Type = Negative, FA = Focused Attention, OM = Open Monitoring, C = Control.
* denotes 95% CI does not contain 0.

**Table 2b.** *Model output of self-compassion moderation for late LPP*						
**Model**	**Fixed Effects**	**Estimate (SD)**	**95% CI**	**R-hat**	**Bulk ESS**	**Tail ESS**
Late LPP	Intercept	0.39 (1.07)	[-1.80, 2.49]	1.01	1182	1659
	Pic Type	0.79 (0.09)	[0.60, 0.97]*	1.00	4265	2988
	Induction FA	0.36 (0.14)	[0.09, 0.62]*	1.00	3724	3222
	Induction OM	-0.25 (0.14)	[-0.51, 0.03]	1.00	3573	3064
	Induction C	-0.11 (0.14)	[-0.38, 0.15]	1.00	3392	3064
	Self-Compassion	-0.24 (0.35)	[-0.92, 0.45]	1.00	1034	1710
	Trait Mindfulness	0.67 (0.36)	[-0.06, 1.40]	1.00	825	1907
	Time	-0.41 (0.08)	[-0.58, -0.25]*	1.00	4245	2739
	Session Number	0.02 (0.12)	[-0.21, 0.24]	1.00	4121	3026
	Task Number	0.83 (0.63)	[-0.43, 2.07]	1.01	1055	1650
	Pic Type:Induction FA	0.21 (0.13)	[-0.04, 0.47]	1.00	3105	3252
	Pic Type:Induction OM	-0.11 (0.13)	[-0.36, 0.15]	1.00	3267	2917
	Pic Type:Induction C	-0.11 (0.13)	[-0.37, 0.16]	1.00	2540	2825
	Pic Type: Self-Compassion	0.21 (0.09)	[0.03, 0.38]*	1.00	4383	3158
	Induction FA: Self-Compassion	0.08 (0.14)	[-0.18, 0.35]	1.00	3548	3194
	Induction OM: Self-Compassion	0.15 (0.14)	[-0.12, 0.41]	1.00	3383	3140
	Induction C: Self-Compassion	-0.23 (0.13)	[-0.49, 0.04]	1.00	3347	2883
	Pic Type:Induction FA: Self-Compassion	0.66 (0.13)	[0.40, 0.92]*	1.00	4037	3493
	Pic Type:Induction OM: Self-Compassion	-0.33 (0.14)	[-0.60, -0.08]*	1.00	3893	3120
	Pic Type:Induction C: Self-Compassion	-0.33 (0.14)	[-0.60, -0.06]*	1.00	3056	2880

Note. Pic Type = Negative, FA = Focused Attention, OM = Open Monitoring, C = Control.
* denotes 95% CI does not contain 0.

Model 3: Moderation by Trait Mindfulness

Early LPP

There was a main effect of Picture Type, such that negative pictures were associated with larger early LPP amplitudes relative to neutral pictures (*b* = 1.64, *sd* = 0.14, 95% CI = [1.38, 1.92]). There were no other main or interactive effects (*b*s < |0.40|, all 95% CIs contain 0). See Table 3a for full model output.

Late LPP

There were main effects of Picture Type and Time, such that negative pictures were associated with larger late LPP amplitudes relative to neutral pictures (*b* = 0.78, *sd* = 0.09, 95% CI = [0.61, 0.96]); and that the late LPP decreased over time *(b* = -0.41, *sd* = 0.85, 95% CI = [-0.57, -0.25]). There was also a main effect of Induction, such that FA was associated with larger LPP amplitudes (*b* = 0.36, *sd* = 0.13, 95% CI = [0.10, 0.62]). The main effect of Picture Type was qualified by a 2-way Picture Type x Trait Mindfulness interaction, such that participants with higher levels of trait mindfulness exhibited larger LPPs on negative picture trials (*b* = 0.51, *sd* = 0.10, 95% CI = [0.31, 0.70]). This two-way interaction was further qualified by a 3-way Trait Mindfulness x Picture Type x Induction interaction, such that participants with higher levels of trait mindfulness exhibited smaller LPPs on negative picture trials during the active control induction (*b* = -0.31, *sd* = 0.13, 95% CI = [-0.56, -0.04]). There were no other main or interactive effects (*b*s < |0.85|, all 95% CIs contain 0). See Table 3b for full model output.

Table 3a. *Model output of trait mindfulness moderation for early LPP*

Model	Fixed Effects	Estimate (SD)	95% CI	R-hat	Bulk ESS	Tail ESS
Early LPP	Intercept	4.88 (1.88)	[1.18, 8.68]*	1.01	926	1311
	Pic Type	1.64 (0.14)	[1.38, 1.92]*	1.00	3862	3061
	Induction FA	0.06 (0.21)	[-0.36, 0.46]	1.00	3295	2687
	Induction OM	-0.11 (0.21)	[-0.53, 0.29]	1.00	2912	2970
	Induction C	0.05 (0.20)	[-0.34, 0.46]	1.00	3032	2591
	Trait Mindfulness	0.18 (0.60)	[-1.01, 1.36]	1.00	1015	1301
	Session Number	-0.17 (0.18)	[-0.51, 0.19]	1.00	4039	2628
	Task Number	0.40 (1.13)	[-1.86, 2.61]	1.01	909	1441
	Pic Type:Induction FA	0.09 (0.21)	[-0.32, 0.49]	1.00	3298	3140
	Pic Type:Induction OM	-0.08 (0.21)	[-0.47, 0.33]	1.00	3226	3125
	Pic Type:Induction C	-0.02 (0.21)	[-0.43, 0.39]	1.00	3287	2908
	Pic Type: Trait Mindfulness	0.16 (0.15)	[-0.12, 0.46]	1.00	3842	2846
	Induction FA: Trait Mindfulness	0.08 (0.21)	[-0.34, 0.49]	1.00	3117	2601
	Induction OM: Trait Mindfulness	-0.27 (0.21)	[-0.70, 0.14]	1.00	3258	2763
	Induction C: Trait Mindfulness	0.19 (0.21)	[-0.21, 0.61]	1.00	2944	2984
	Pic Type:Induction FA: Trait Mindfulness	0.07 (0.21)	[-0.33, 0.49]	1.00	2755	2619
	Pic Type:Induction OM: Trait Mindfulness	0.11 (0.21)	[-0.29, 0.52]	1.00	2789	2764
	Pic Type:Induction C: Trait Mindfulness	-0.18 (0.21)	[-0.58, 0.24]	1.00	2800	3028

Note. Pic Type = Negative, FA = Focused Attention, OM = Open Monitoring, C = Control.
* denotes 95% CI does not contain 0.

Table 3b. *Model output of trait mindfulness moderation for late LPP*

Model	Fixed Effects	Estimate (SD)	95% CI	R-hat	Bulk ESS	Tail ESS
Late LPP	Intercept	0.42 (1.11)	[-1.74, 2.62]	1.02	743	1272
	Pic Type	0.78 (0.09)	[0.61, 0.96]*	1.00	4297	3006
	Induction FA	0.36 (0.13)	[0.10, 0.62]*	1.00	3480	3256
	Induction OM	-0.25 (0.13)	[-0.50, 0.01]	1.00	3402	2937
	Induction C	-0.11 (0.13)	[-0.38, 0.15]	1.00	3149	2831
	Trait Mindfulness	0.56 (0.33)	[-0.08, 1.21]	1.00	755	1654
	Time	-0.41 (0.85)	[-0.57, -0.25]*	1.00	4553	2843
	Session Number	-0.01 (0.12)	[-0.23, 0.22]	1.00	3832	3099
	Task Number	0.85 (0.66)	[-0.50, 2.13]	1.02	595	1224
	Pic Type:Induction FA	0.22 (0.13)	[-0.04, 0.49]	1.00	3773	3251
	Pic Type:Induction OM	-0.12 (0.13)	[-0.37, 0.13]	1.00	3954	3363
	Pic Type:Induction C	-0.10 (0.13)	[-0.36, 0.16]	1.00	3382	2961
	Pic Type:Trait Mindfulness	0.51 (0.10)	[0.31, 0.70]*	1.00	5149	2937
	Induction FA: Trait Mindfulness	0.16 (0.14)	[-0.11, 0.43]	1.00	2871	2708
	Induction OM: Trait Mindfulness	-0.15 (0.14)	[-0.41, 0.11]	1.00	2918	3048
	Induction C: Trait Mindfulness	-0.01 (0.13)	[-0.27, 0.25]	1.00	3193	3334
	Pic Type:Induction FA: Trait Mindfulness	0.10 (0.14)	[-0.17, 0.37]	1.00	2836	2668
	Pic Type:Induction OM: Trait Mindfulness	0.21 (0.14)	[-0.06, 0.47]	1.00	3237	2918
	Pic Type:Induction C: Trait Mindfulness	-0.31 (0.13)	[-0.56, -0.04]*	1.00	3521	2836

Note. Pic Type = Negative, FA = Focused Attention, OM = Open Monitoring, C = Control.
* denotes 95% CI does not contain 0.

References Cited

1. Baer RA, Smith GT, Hopkins J, Krietemeyer J, Toney L (2006): Using self-report assessment methods to explore facets of mindfulness. *Assessment* 13: 27–45.
2. Brown KW, Ryan RM (2003): The benefits of being present: Mindfulness and its role in psychological well-being. *Journal of Personality and Social Psychology* 84: 822–848.
3. Lin Y, White ML, Viravan N, Braver TS (2024): Parsing state mindfulness effects on neurobehavioral markers of cognitive control: A within-subject comparison of focused attention and open monitoring. *Cogn Affect Behav Neurosci*. https://doi.org/10.3758/s13415-024-01167-y
4. Watson D, Clark LA, Tellegen A (1988): Development and validation of brief measures of positive and negative affect: The PANAS scales. *Journal of Personality and Social Psychology* 54: 1063–1070.
5. Hoddes E, Dement W, Zarcone V (1972): The development and use of the Stanford Sleepiness Scale (SSS). *Psychophysiology* 9: 150.
